# Steady-State Visual Evoked Potential-Based Brain–Computer Interface Using a Novel Visual Stimulus with Quick Response (QR) Code Pattern

**DOI:** 10.3390/s22041439

**Published:** 2022-02-13

**Authors:** Nannaphat Siribunyaphat, Yunyong Punsawad

**Affiliations:** 1 School of Informatics, Walailak University, Nakhon Si Thammarat 80160, Thailand; nannaphat.si@mail.wu.ac.th; 2 Informatics Innovative Center of Excellence, Walailak University, Nakhon Si Thammarat 80160, Thailand

**Keywords:** brain-computer interface, electroencephalography, steady-state visual evoked potential (SSVEP), quick response, QR code, visual fatigue

## Abstract

Steady-state visual evoked potential (SSVEP)-based brain-computer interface (BCI) systems suffer from low SSVEP response intensity and visual fatigue, resulting in lower accuracy when operating the system for continuous commands, such as an electric wheelchair control. This study proposes two SSVEP improvements to create a practical BCI for communication and control in disabled people. The first is flicker pattern modification for increasing SSVEP response through mixing (1) fundamental and first harmonic frequencies, and (2) two fundamental frequencies for an additional number of commands. The second method utilizes a quick response (QR) code for visual stimulus patterns to increase the SSVEP response and reduce visual fatigue. Eight different stimulus patterns from three flickering frequencies (7, 13, and 17 Hz) were presented to twelve participants for the test and score levels of visual fatigue. Two popular SSVEP methods, i.e., power spectral density (PSD) with Welch periodogram and canonical correlation analysis (CCA) with overlapping sliding window, are used to detect SSVEP intensity and response, compared to the checkerboard pattern. The results suggest that the QR code patterns can yield higher accuracy than checkerboard patterns for both PSD and CCA methods. Moreover, a QR code pattern with low frequency can reduce visual fatigue; however, visual fatigue can be easily affected by high flickering frequency. The findings can be used in the future to implement a real-time, SSVEP-based BCI for verifying user and system performance in actual environments.

## 1. Introduction

The cause of disability can be a genetic disorder, congenital illness, accident, or unknown. Disabilities have different symptoms and levels of severity. One of the major problems is the inability to move, which results in dependence on mobility equipment for assistance in everyday life. So far, many assistive devices have been developed. Yet, they cannot cover all levels of disabilities, especially for severely paralyzed patients who completely lose movement and communication abilities [1]. As a result, they require advanced assistive technology, through employing biomedical signals to directly interface with the machine or device [2].

A brain–computer interface (BCI) is an emerging human-computer interaction (HCI) technology used to communicate between the human brain and computers [3,4]. A BCI can create a replacement or alternative pathway connection between the brain and prostheses or assistive devices for spinal cord injury (SCI), stroke, and amyotrophic lateral sclerosis (ALS) (also known as neuroprosthetics). Generally, the three main parts of a BCI consist of (1) brain signals and data acquisition, (2) a feature extraction and classification algorithm, and (3) command translation and applications. A non-invasive BCI [5] is a popular technique for research and development. A practical BCI system is the primary goal of a non-invasive BCI that requires non-contact and contact sensors for signal acquisition, such as electroencephalography (EEG) [6] and near-infrared (NIR) machines [6]. An EEG is a popular brain signal acquisition system for measuring and recording phenomena that occur rapidly and require high resolution and sensitivity to changes in phase, such as evoked potentials (EPs), transient responses to sensory stimulation. Additionally, an EEG-based BCI system [3] can be developed for practical, portable EEG devices that have been designed to facilitate their use through dry electrodes, without conductive gel or saline solution. For medical applications, an EEG-based BCI is usually employed for assistive technology, neurotherapy [7], detection, diagnosis [8], rehabilitation, and restoration [5,9]. Besides, non-medical applications, consisting of entertainment, games [10], industry [11], and transport [12], have been applied.

Normally, EEG signals are spontaneous brain potentials and event-related potentials (ERPs) to generate BCI commands. Event-related desynchronization (ERD) or synchronization (ERS) occur from mental imagery paradigms, such as motor and speech imagination [13,14]. Moreover, time- and phase-lock phenomena happen from an external stimulus through the sensory nerve and, especially, the visual system that usually applies different stimulus patterns or paradigms to activate neurons of the visual cortex at the occipital lobe. EEG signals are measured using visual stimulation as visual evoked potentials (VEPs). VEPs can be divided into two types, based on different techniques of visual stimulus: (1) transient VEP or P300 [15] and (2) steady-state visual evoked potential (SSVEP) [16]. The SSVEP is a brain signal, which has steady periodic to visual stimulation, with a specific frequency. When the optic nerve is stimulated at a frequency in the range of 3.5–75 Hz, the brain generates an electrical signal of the same frequency or multiple frequencies; this can be stimulated by a light-emitting diode (LED), image on a liquid crystal display (LCD), animation, or pattern image [17]. A SSVEP-based BCI is widely used for electric wheelchairs. Previously, many researchers demonstrated that SSVEP achieves high accuracy, high information transfer rate (ITR), and less time for user training than other BCI techniques [18]. However, SSVEP-based BCI systems have the following weaknesses: (1) users can experience visual fatigue from focusing and attending flicker stimulus patterns over an extended period of time, and (2) the SSVEP response is still unclear for some users who can perform with low or high visual stimulus pattern flickering frequency. Both are challenging for practical applications, and many research groups have attempted to investigate methods for improving SSVEP-based BCIs.

Generally, the use of BCI systems can cause mental fatigue [19]. In addition, the VEP-BCI system induces visual fatigue through the visual stimuli. SSVEP makes visual fatigue easier than the P300 method, through visual stimulus patterns and paradigms. Visual fatigue directly affects the user and system performances; it can make users uncomfortable when focusing on visual stimuli for long periods of time and, consequently, decrease accuracy [20]. To reduce and prevent visual fatigue, visual stimulators have been improved, based on human vision and perception conditions, i.e., color, shape, luminance (display devices), flicker frequency (phase), and pattern (checkerboard and motion onset). The aim of the improvements is that the user can yield a strong SSVEP response immediately after the stimulus with less visual fatigue; this can be utilized in continuous BCI command creation, such as control of an electric wheelchair or prosthetic arm.

Moreover, a combination with other BCI methods has also been demonstrated to replace SSVEP during visual fatigue [21,22]. However, the other BCI system incurs high cost and increases the complexity for the user. Therefore, designing a novel visual stimulus pattern is a popular approach for enhancing and improving the SSVEP technique. Hence, this study proposes a novel visual stimulus pattern to improve SSVEP-BCI performance.

According to human perception, light is reflected on the object and comes through the retina, which contains two photoreceptors, i.e., rods and cones. The rod cells are more sensitive to light and dark changes, shape, and movement. Cones are more sensitive to green, red, or blue colors, but less sensitive to light than rod cells because almost all previous research studies use a black and white checkerboard for visual stimuli. Some researchers have explored different colors and shades for the SSVEP-BCI system by benchmarking the maximum amplitude of the power spectrum at the flickering frequency [23,24,25,26,27,28]. However, the results cannot validate the SSVEP response or which color or shade is suitable for all users, and more verification of light intensity and flicker pattern is required, in order to reduce visual fatigue.

Studies of SSVEP visual stimulation are interesting for developing a conventional visual stimulus and investigating novel patterns to modulate direct responses in the visual cortex. For example, Waytowich et al. [25] studied the enhancement of SSVEP stimulation from nine chessboard patterns. The spatial frequency of 2.4 cycles per degree (32 × 32) can increase the maximum data transfer rate and reduce eye irritation, compared to the lower spatial frequency. Furthermore, SSVEP stimulation, with image modification and characteristic detection algorithms, was studied to design the coding frequency and phase, in order to solve harmonic frequency problems in the SSVEP-BCI system. The results showed that the classification rate increased by more than 10% [29]. In addition, Keihani et al. [26] optimized the rate of fatigue when participants were exposed to high-frequency sine wave stimulation, with LEDs from three frequencies (25, 30, and 35 Hz), to determine the pattern with the lowest fatigue rate. The sorting of the excitation frequency in the 35-35-35 Hz sequence has the lowest fatigue rate from the obtained results.

In sequences contrast, the 25-25-25 Hz sequence exhibited the highest rate of fatigue. Furthermore, the color of the SSVEP pattern was employed to reduce visual fatigue. The white color gives the best performance, followed by gray, red, green, and blue [23]. Moreover, color-based stimuli were examined by Duart et al. [24]. They carefully verified the use of white, red, and green colors for SSVEP stimuli, since there were previous studies [23] that considered their effects at low (5 Hz), middle (12 Hz), and high (30 Hz) frequencies. They also found that white color has good results, similar to red color, that affect stimulation. The experiments were conducted at the first and second harmonic frequencies. The results showed that in the low frequency range, green and red were suitable for low frequencies. White and red are appropriate for the medium frequencies. For high frequencies, there was no difference among the three colors.

Furthermore, some researchers demonstrated different flicker patterns through employing rapid object motion and lower light intensity than the flashing pattern [30]. For example, the use of object motion or spin patterns [31] was used to activate some phenomena in the visual cortex. This technique can reduce visual fatigue problems but has a low ITR. A SSVEP-based BCI for controlling an electric wheelchair requires a high ITR. A summary of the previous research studies on visual stimuli for SSVEP-based BCI is listed in Table 1, along with some of their proposed methods, visual stimuli, electrode positions, and results. Therefore, a hybrid BCI [21,22,32,33,34] is an alternative technique for obtaining a practical BCI; it is usually developed by combining two BCI modalities, or BCI combined with other HCI modalities, such as electrooculography (EOG) and electromyogram (EMG) signals. In addition, we can combine BCI modalities with external intelligent devices. However, hybrid BCIs can increase the complexity of user command creation.

In this paper, we propose a new visual stimulus pattern for enhancing a SSVEP-based BCI system. The proposed method consists of utilizing a quick response (QR) code to reduce visual fatigue and time for staring. In addition, we proved a SSVEP stimulus by mixing the fundamental frequency and its harmonics for explicit features and mixing two fundamental frequencies to increase commands. We further demonstrate the use of the proposed method, in order to implement a real-time SSVEP-BCI.

## 2. Materials and Methods

### 2.1. EEG Acquisition

Twelve healthy volunteers (seven females and five males, average age of 27.6 ± 2.3 years old) participated in the experiments. All participants have the normal vision acuity for inclusion criteria, without color blindness and neurological disorders (in the past or present). The exclusion criteria were related to participants that had migraines that were activated from visual perception. Before signing a consent form, all participants were informed and read the documentation to participate in the experiment. All signed consent forms were kept confidential (without personal identification). All protocols involving human participants were approved by the Office of the Human Research Ethics Committee of Walailak University, which adopted the Ethical Declarations of Helsinki, Council for International Organizations of Medical Sciences (CIOMS), and the World Health Organization (WHO) guidelines.

**Figure 1:**
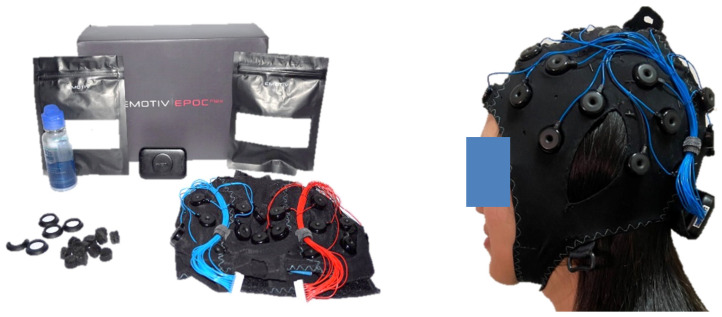
EPOC Flex 32-channel wireless EEG device.

**Figure 2:**
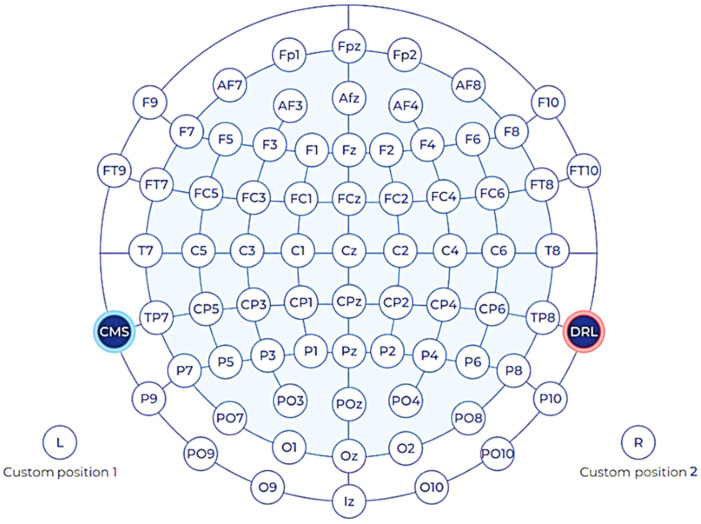
Electrode placement for 32 channels, based on a 10–20 system.

First, we used the 32-channel EPOC Flex^TM^ (shown in Figure 1) from EMOTIV (https://www.emotiv.com, accessed on 16 October 2021), at a sampling rate of 128 Hz. The EPOC Flex is a wireless EEG machine with flexible traditional EEG head cap systems that minimizes the setup time. It measures the electrical brain potentials via saline electrodes and saline soaked felt pads, while it is flexible and easy to use. We also checked that (and adjusted) each electrode’s position placed on the right area. EEG signals were collected using Emotiv Pro [ver. 3.1.3]. We explored the brain response from whole EEG electrodes using topographic brain mapping, in order to verify the proposed visual stimulus patterns. Moreover, the specific EEG electrode positions of interest are around the occipital and parietal areas [18], i.e., PO3, PO4, POZ, O1, O2, and Oz, as shown in Figure 2, for practical BCI systems with a single or a few electrodes.

### 2.2. Proposed Visual Stimulation

The main objective of this work, regarding SSVEP-based BCI systems, is the development of a visual stimulation method to improve the conventional technique. Two main approaches were mixing flicker frequencies and a novel visual stimulus pattern, inspired by the QR code style. Three fundamental frequencies, namely 7, 13, and 17 Hz (Table 2), were selected, based on the previous study on a real-time, SSVEP-based BCI system [35], to verify the proposed visual stimulation pattern. The dimensions of the visual stimuli were 4 cm × 5 cm. The distance between the two stimuli, measured from the center, was 10 cm in the horizontal direction and 5 cm in the vertical direction. The fixation point was located in the middle of the resting state and EEG baseline calibration. Visual stimuli were displayed on a 21.5-inch LED monitor, with a frequency of 75 Hz and a resolution of 1920 × 1080 pixels.

#### 2.2.1. Proposed Mixing of Flicker Frequencies

According to previous research, since a SSVEP response is not solid and transparent for all users, we cannot summarize the low- or high-frequency flickers that are suitable for SSVEP stimulation. Hence, we attempt to provide the SSVEP visual stimulation pattern that cover all users who yield a strong response, with different ranges of flicker frequency. Mixing of flicker frequencies can be divided into two designs: (1) mixing between fundamentals with their harmonic frequencies and (2) mixing between two fundamental frequencies, as shown in Table 2. In the pilot study presented mixing fundamentals with their harmonic frequencies was used, and a small number of subjects performed the SSVEP task [36]. We employed traditional SSVEP feature extraction to verify the proposed visual stimulus. The results showed that the modified flicker visual stimulus could generate more than two dominant frequencies at the 7 and 13 Hz stimulus flickering frequencies, i.e., the sub-harmonic, first harmonic, and second harmonic frequencies, for all subjects. Moreover, this work proposed an increasing number of commands; we observed mixing two fundamental frequencies. The three flicker patterns, consisting of 7 and 13 Hz, 7 and 17 Hz, and 13 and 17 Hz, are shown in Table 2 (flicker patterns 6, 7, and 8).

#### 2.2.2. Proposed Visual Stimulation Using QR Code Patterns

**Figure 3:**
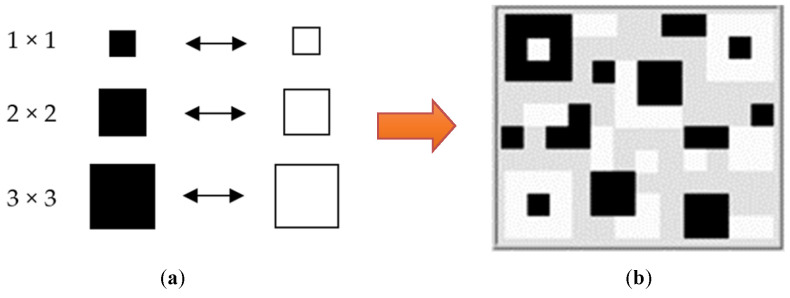
Proposed SSVEP stimulation pattern. (**a**) Three different sizes of the components inside stimulation pattern (1:10 mm). (**b**) Example of stimulation pattern using the QR code style.

**Figure 4:**
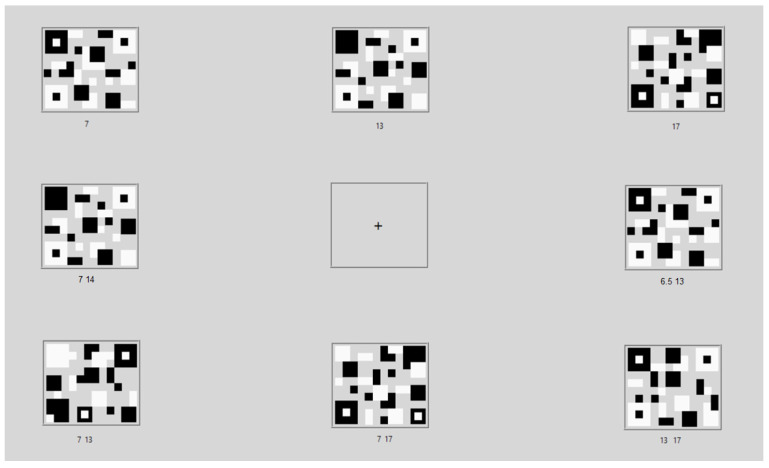
Screenshot the proposed visual stimuli, using a QR code pattern with three fundamental flickering frequencies and harmonics for eight different flicker patterns (Table 2), through an LCD monitor for SSVEP stimulation.

**Figure 5:**
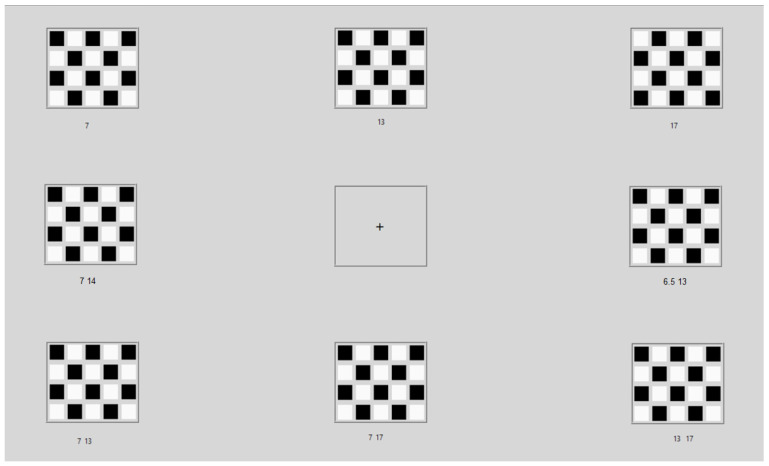
Screenshot conventional visual stimuli, using a checkerboard pattern with three fundamental flickering frequencies and harmonics for eight different flicker patterns (Table 2), through an LCD monitor for SSVEP stimulation.

According to conventional visual stimuli, using a checkerboard pattern, a square shape with the same size and position is usually used. We noticed that QR code patterns, related to the checkerboard, could be employed for visual stimulus patterns. A QR code is a two-dimensional matrix barcode [37], invented by the Japanese automotive company Denso Wave. The representation of the QR code pattern was adopted for the visual stimulus pattern. We attempted to observe a stimulus pattern, consisting of three different squares with random locations, to induce visual evoked potentials. The proposed patterns were implemented using the LabVIEW program, as shown in Figure 3. For the SSVEP stimulator, each proposed pattern was located around the screen border, as shown in Figure 4. Eight different stimulus patterns and flickering frequencies, as shown in Table 2, were used to explore the SSVEP response between the proposed and conventional visual stimulus patterns, as shown in Figure 5. We expected that the proposed visual stimulus pattern would produce a more explicit and permanent SSVEP than the checkerboard pattern; moreover, it may reduce visual fatigue.

### 2.3. SSVEP Detection Methods

MATLAB (MathWorks) [ver. R2019a] was used to process and analyze the recorded EEG signals from each participant. A 50 Hz notch filter was used to remove power line noise, and a 3–40 Hz bandpass digital filter was used to avoid motion artefacts. For SSVEP detection, power spectral density (PSD) and canonical correlation analysis (CCA) were used to analyze the effects of brain signals [38], as described in the following subsections.

#### 2.3.1. Power Spectral Density (PSD)

The PSD method uses the power distribution of electrical brain signals in the frequency domain to apply the results to commands or decision-making. It looks at changes in density in different regions of the brain, focusing on how the stimulus area changes [39,40]. This study focused on the stimulation of regions in the occipital cortex, activated by visual perception, to determine the relationship between the target stimuli and brain signal modulation. The Welch algorithm was used to estimate the PSD by separating signals into windows of the same size. The Fourier transform is calculated on each segment, resulting in the squared value. Then, we calculated the average of all periodograms, which was calculated as a PSD estimation [41]. For implantation, the ‘pwelch’ function was used to determine the PSD, using a Hamming window with 50% overlapping. It returns an estimate of the Welch PSD, at the frequency specified, to compare with the baseline from the resting period for SSVEP response detection.

#### 2.3.2. Canonical Correlation Analysis (CCA)

CCA is a popular statistical method for analyzing brain signals. CCA is widely used in SSVEP target detection. CCA can assess the relationship between the data to be examined with the previously defined reference (sinusoidal signals at flickering frequencies), in order to find the canonical correlation values [42]. The maximum target of the correlation coefficient was selected to identify the target frequency of brain signals used to generate commands for the BCI, in order to control devices or make decisions about answer choices. In this study, CCA was applied to examine brain stimulation from checkerboard patterns and QR code pattern stimuli, by the SSVEP method, for validity. We expect it to be able to correctly identify the stimulus signal, since it focuses on brain signals in areas related to vision.

### 2.4. Experiments

**Figure 6:**
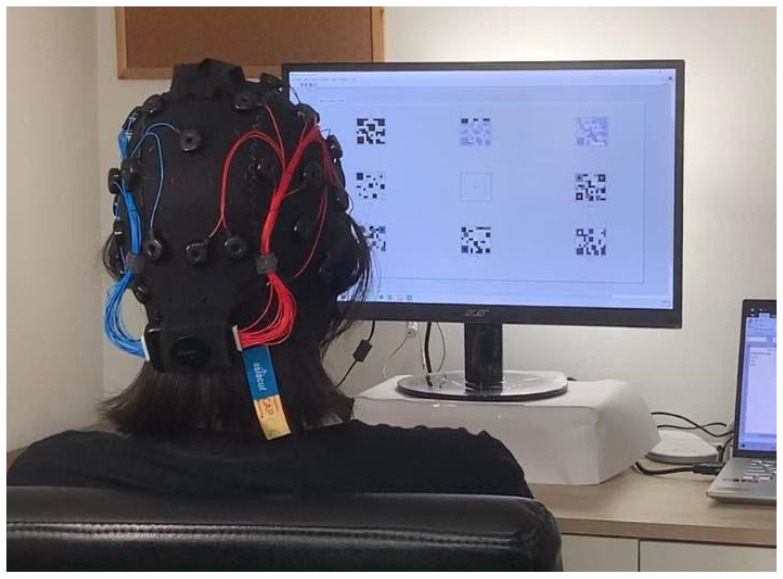
Experimental setup during QR code flickering pattern stimulation.

The experiment was conducted in a quiet room with a typical indoor light environment. Participants sat in front of the LCD monitor, at a distance of 60 cm, as shown in Figure 6. All flicker patterns on the screen were displayed to the participant simultaneously during the QR code pattern and checkerboard pattern experiments, as shown in Figure 4 and Figure 5, respectively. Each participant stared at the flicker by following the sequence in Table 2 (20 times for each pattern) through two visual stimulus patterns, starting with the QR code pattern (Figure 4). Each time consisted of a resting period of 5 s and stimulus period of 5 s. After finishing every flicker, the subject rested for 5 min before starting the next flicker. Before moving to the checkerboard pattern (Figure 5), the participant rested for 10 min. Moreover, participants were scored with levels of 1 to 5, for the visual fatigue questionnaire, by following the visual analogue scale-based pain measurement to assess the participant’s feelings of visual comfort to the QR code and checkerboard stimulus patterns (with different flicker stimuli). All participants received instruction on the visual fatigue scoring system, which has five levels, as follows: 1 indicates comfortable, 2 indicates rather comfortable, 3 indicates mildly uncomfortable, 4 indicates rather uncomfortable, and 5 indicates highly uncomfortable.

### 2.5. Observation of SSVEP Responses from QR Code Flickering Pattern Stimulation

**Figure 7:**
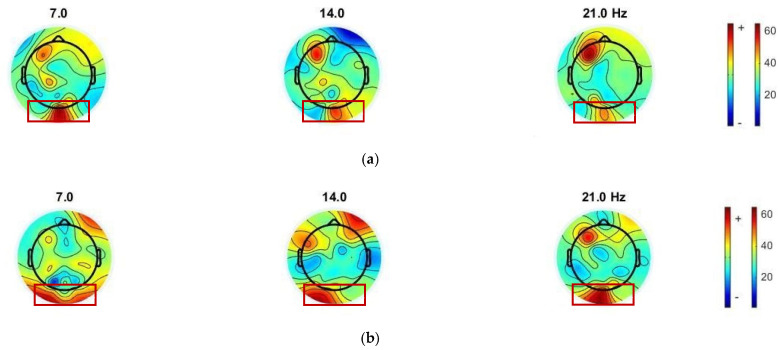
Topographic brain mapping of SSVEP responses of participant 3: (**a**) SSVEP visual stimulation, using the checkerboard pattern and mixing fundamental flicker frequency at 7 Hz and harmonic frequency at 14 Hz; (**b**) SSVEP visual stimulation, using the QR code pattern and mixing fundamental flicker frequency at 7 Hz and harmonic frequency at 14 Hz.

**Figure 8:**
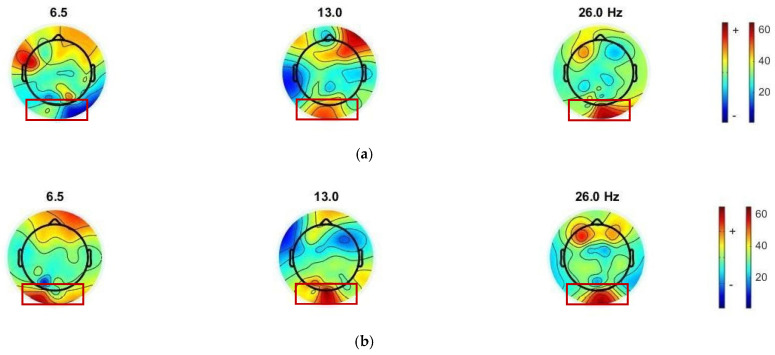
Topographic brain mapping of SSVEP responses of participant 3: (**a**) SSVEP visual stimulation, using the checkerboard pattern and mixing fundamental flicker frequency at 13 Hz and sub-harmonic frequency at 6.5 Hz; (**b**) SSVEP visual stimulation, using the QR code pattern and mixing fundamental flicker frequency at 13 Hz and sub-harmonic frequency at 6.5 Hz.

To observe the SSVEP response, the EEGLAB toolbox [43] was used to generate a topographic brain mapping from the PSD, in order to visualize the SSVEP features between conventional and proposed visual stimulus patterns. We visually observed each type of visual stimulus pattern, according to the topographic mapping of the average normalized power (shown in Figure 7 and Figure 8). The occipital response, i.e., an area of the SSVEP stimulation response, was observed. The electrode positions of interest in the occipital region were PO3, PO4, POZ, O1, O2, and Oz.

The topographic brain mapping demonstrated a high-intensity PSD in the occipital area. For the mixing of 7 and 14 Hz stimuli via the QR code pattern (Figure 7b), we observed that the intensity of PSD in the occipital area exhibited a more significant response at fundamental and harmonics, at 7, 14, and 21 Hz, from the checkerboard pattern (Figure 7a). For the mixing of 13 and 6.5 Hz stimulus via the QR code pattern (Figure 8b), we observed that the intensity of PSD in the occipital area exhibited more significant responses, at 6.5, 13, and 26 Hz, from the checkerboard pattern (Figure 8a).

Each participant may have a dominant SSVEP frequency at different flickering frequencies, i.e., exactly at the stimulus frequency, first harmonic, second harmonic, or sub-harmonic. However, after averaging the waveforms from all subjects, we can still observe the activation of these target frequencies by using a well-known method to detect a SSVEP response, such as the PSD and CCA methods, based on this experiment. Low or high frequencies can be used for our proposed stimulus patterns, and they vary for different users.

## 3. Results

**Figure 9:**
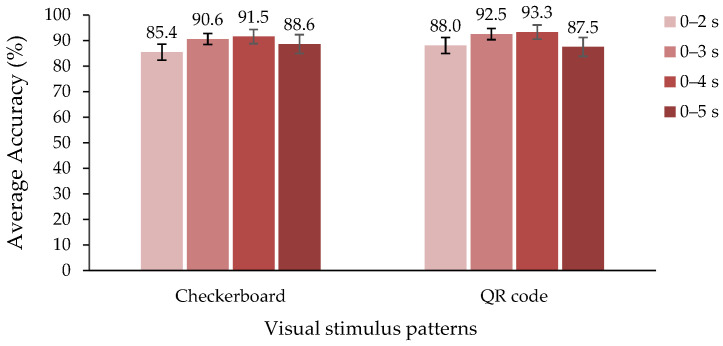
SSVEP stimulus duration of the checkerboard and QR code patterns, using PSD and CCA classification confidence interval (alpha: 0.01).

To verify the stimulus duration for SSVEP detection from two different SSVEP stimulus patterns, we used four windows, with 50% overlap, to detect the dominant frequencies, including 2, 3, 4, and 5 s PSD and CCA methods. Less time for visual stimuli can reduce visual fatigue. Hence, we attempted to observe the time and SSVEP classification accuracy between the checkerboard and QR code stimulus patterns for efficiency and visual fatigue evaluation. According to Figure 9, we found that 3 and 4 s, with both stimulus patterns, can provide a high efficiency of the SSVEP methods. The minimum time for SSVEP stimulation was approximately 3–4 s, and the average classification accuracy ranged from 90.6% to 93.3%. The QR code yielded a higher efficiency than the checkerboard pattern for 2 to 4 s. A decrease in classification accuracy at 5 s can occur from a reduced SSVEP intensity at 4–5 s, since an extended period of stimulus can lead to visual fatigue.

### 3.1. Evaluation of Mixing Flicker Frequencies

According to the results in Table 3, three issues of the proposed SSVEP stimulus are listed. The first is the efficiency of the proposed visual stimulus, when using the QR code pattern. The average accuracy of the checkerboard pattern ranged from 83.7% to 90.9% for all flicker patterns, and the QR code pattern ranged from 87.3% to 94.4%, which was higher than the checkerboard pattern. The second is an evaluation of mixing flickers between fundamental and harmonic frequencies (flicker patterns 4 and 5, at 7–14 Hz and 6.5–13 Hz, respectively) for comparison with the conventional flicker using only the conventional fundamental frequency (flicker patterns 1 and 2, at 7 Hz and 13 Hz, respectively) for the SSVEP stimulus. The average classification accuracy of using only the fundamental frequency ranged from 84.9% to 91.8%. The average accuracy of the mixing flickers between the fundamental and harmonic frequencies ranged from 83.7% to 94.4%. We found that both flickers provided similar efficiencies. The third issue considers command increments by mixing two fundamental frequencies, i.e., flicker patterns 6, 7, and 8. The average classification accuracy of the checkerboard pattern ranged from 84.7% to 90.5%, and the QR code pattern ranged from 88.0% to 93.0%. PSD methods can provide a high average accuracy for SSVEP classification by mixing two fundamental frequencies, and the maximum frequency was 93.2% from pattern six, through the QR code pattern.

**Figure 10:**
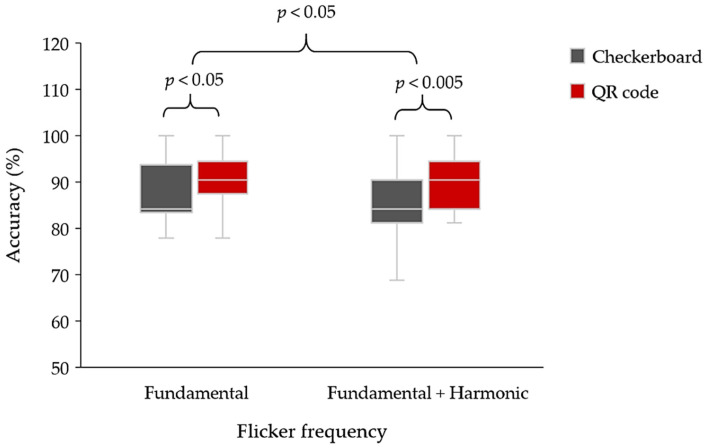
Average classification accuracy between using only the fundamental (flicker pattern 1 and 2) and mixing fundamental and harmonic frequencies (flicker pattern 4 and 5) of the checkerboard and QR code patterns of SSVEP stimulus (shown in Table 3).

Furthermore, there is a significant difference between using only the fundamental (conventional) and mixing fundamental and harmonic frequencies (proposed); the effect of each pattern was inspected (shown in Figure 10). The paired t-test for the mean was used to analyze a statistically significant difference between the groups of flicker stimuli (Table 2) and visual stimulus patterns. First, the paired *t*-test (*n* = 24) indicated that there was a significant difference between the average classification accuracy of using only the fundamental frequency of the checkerboard and QR code pattern (*p =* 0.010; *p* < 0.05). The paired *t*-test (*n* = 24) also indicated a statistically significant difference between the accuracy of mixing the fundamental and harmonic frequencies of the checkerboard and QR code patterns (*p* = 0.001; *p* < 0.005). Second, the paired *t*-test (*n* = 48) indicated a statistically significant difference between the average classification accuracy of using only the fundamental and mixing fundamental and harmonic frequencies (*p* = 0.033; *p* < 0.05).

### 3.2. Evaluation of QR Code Pattern as SSVEP Stimulus

According to Table 4, the average classification accuracy of the checkerboard pattern ranged from 83.6% to 91.3%, while the average classification accuracy of the QR code pattern ranged from 85.9% to 94.4%. The maximum classification accuracy of the PSD method was 93.0%, while the maximum classification accuracy, using the CCA method for SSVEP detection, was 94.4% for the QR code visual stimulus pattern. The results verified and supported the proposed visual stimulus pattern. The QR code pattern with the CCA method can generate the average classification accuracy of all participants. For some participants, both stimulus patterns gave similar classification accuracy for each SSVEP detection method.

**Figure 11:**
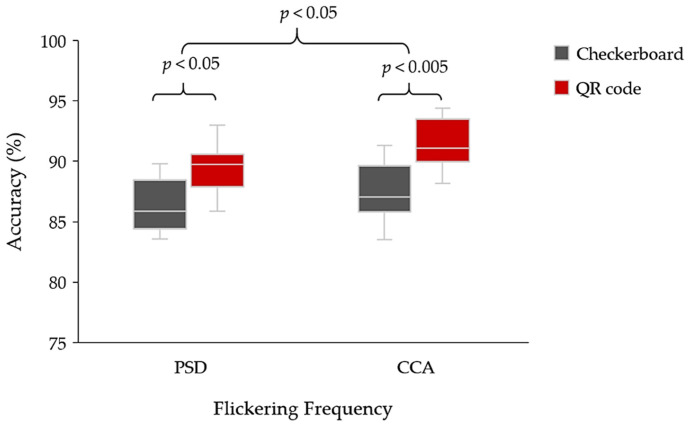
Average classification accuracy between the checkerboard and QR code patterns of SSVEP stimulus for the PSD and CCA methods.

According to Figure 11, two main issues were identified. The first issue regards the SSVEP detection method for visual stimulus patterns. The paired *t*-test (*n* = 24) reported a statistically significant difference between the PSD and CCA methods (*p* = 0.012; *p* < 0.05). Additionally, CCA can yield a higher efficiency than the PSD method. The second issue is the efficiency of each type of visual stimulus pattern. The paired *t*-test (*n* = 12) indicated a significant difference between the QR code and checkerboard patterns, using the PSD method (*p* = 0.013; *p* < 0.05). Furthermore, the paired t-test indicated a significant difference between the QR code and checkerboard patterns, using the CCA method (*p* = 0.001; *p* < 0.005). Even though the proposed SSVEP visual stimulus via QR code pattern can achieve a higher average classification accuracy than the checkerboard pattern for all participants, visual fatigue must be observed between the QR code and checkerboard patterns, for practical purposes. The visual fatigue issue will be described in Section 3.3.

### 3.3. Visual Fatigue

**Figure 12:**
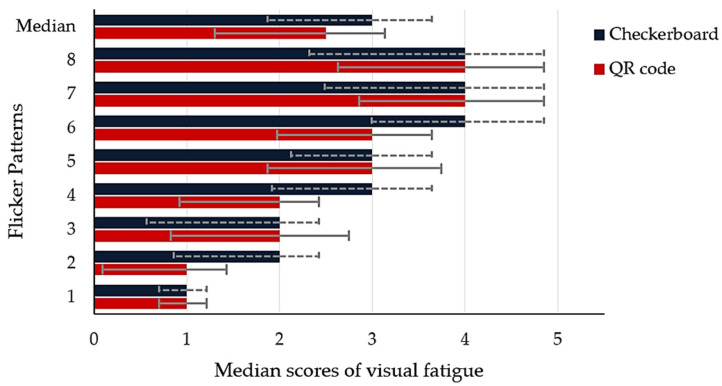
Visual fatigue scores from all participants, after performing the QR code and checkerboard stimulus patterns, with different flicker stimuli, with 95% confidence intervals.

After the experiment, participants were asked about their visual fatigue score for each stimulus (Table 2), affecting their eyes on a scale from 1 to 5, with 1 indicating minimum visual fatigue and 5 indicating maximum visual fatigue. The median is used as a robust measure to conceal higher levels of variability in the visual fatigue score. The results are presented in Figure 12. Flicker patterns 4 to 8 (mixing of flicker frequency) had a more significant effect on the eyes (three to four scores), rather than flicker patterns 1 to 3 (only fundamental frequency), which can provide only one to two scores for both patterns. In contrast, the proposed mixing flickering frequency can yield higher SSVEP responses than the conventional method. Moreover, a comparison of visual fatigue between the QR code and checkerboard patterns showed that the QR code pattern had a median score of 2.5, while the checkerboard pattern had 3. However, there was no significant difference between the QR codes and checkerboard patterns, even though nine out of twelve participants (75%) recommended the QR code pattern for less visual fatigue.

## 4. Discussion

To investigate the proposed SSVEP visual stimuli via a QR code pattern for BCIs, topographic brain mapping of the QR code stimulus pattern illustrated the SSVEP response of the occipital area. In addition, we modified the flicker patterns. The experiment revealed that mixing fundamentals, and their harmonic frequencies (Table 2), provide a better SSVEP response than the fundamental frequency for stimulus pattern and testing of SSVEP detection methods alone. Furthermore, both PSD and CCA methods yielded acceptable accuracies.

Moreover, the traditional SSVEP can create three additional commands by utilizing the proposed mixing fundamental frequencies. The experiment also revealed additional details of designing the SSVEP visual stimulus pattern through employing a QR code format, including the size and flickering frequencies of the pattern, to reduce eye fatigue; the QR code is recommended. The classification accuracy of the QR code pattern may slightly increase, compared to that of the checkerboard, and visual fatigue of low flickering frequency will decrease, according to the participants’ opinions. However, the proposed visual stimulation required more than 2 s to achieve a high efficiency of SSVEP detection, which is similar to the conventional and previous SSVEP systems [27,35].

Finally, some limitations of the modified flicker and QR code patterns for SSVEP stimulation can be reported. First, after the initial verification of the modified flicker (mixing frequencies) for the SSVEP stimulus with a small number of frequencies, we aim to further observe additional fundamental frequencies. Secondly, some subjects reported visual fatigue when using the QR code pattern with a high flickering frequency. Hence, we have to further validate the sizes and shapes (circle) of the components inside the QR code patterns. Lastly, the ITR should be verified for real-time, SSVEP-based BCI systems.

## 5. Conclusions

This study proposes a new SSVEP visual stimulus, with a QR code pattern for an EEG-based BCI system. Furthermore, we proposed a mixing of flickering frequency stimulation patterns with the recommended 7, 13, and 17 Hz flicker frequencies and created eight commands. The SSVEP responses from the topographic brain mapping of the proposed visual stimuli were investigated. The mixing of flicker frequencies can be efficiently used to enhance the SSVEP response and increase the number of commands from the conventional SSVEP-based BCI. Furthermore, we conclude that the QR code pattern is an efficient stimulus pattern that can be used for SSVEP stimulation. Both PSD and CCA methods can be used for SSVEP detection of the proposed QR code stimulation pattern. The proposed SSVEP stimulus patterns can be employed to enhance a real-time, SSVEP-based BCI system, for practical use in people with disabilities. The proposed system can be implemented in SSVEP-based BCI systems for powered wheelchair control and spellers. We suggest that the proposed SSVEP stimulus, via QR code pattern, can be further explored to reduce time and visual fatigue by using random location of the piece of the QR pattern to generate evoked potentials.

## Figures and Tables

**Table 1:** Research studies on visual stimuli for SSVEP-based BCI.

Authors	Proposed Method	Visual Stimuli	Electrode Positions	Result(s)
Duart et al., 2020 [24]	Effect of stimuli color and frequency	Red, green, and white color with 5, 12, and 30 Hz frequencies on the auxiliary display.	PO3, PO4, Pz, O1, O2, Oz	- Red and white can be used at medium frequency. - Green and red can be used at low frequency. - No difference between colors at high frequencies.
Waytowich et al., 2016 [25]	Optimization of checkerboard spatial frequencies	Solid background to single-pixel checkerboard pattern with 2.4 cycles per degree.	Oz, O1, O2, POz, PO3, PO4, PO7, PO8	Spatial frequency can have a dramatic effect on SSVEP performance that is consistent across subjects
Keihani et al., 2018 [26]	3-sequence frequency	LED and three-fiber optic sensor with high frequencies (25, 30, and 35 Hz).	O1, Oz, O2	Accuracy rate for PSD was 88.35% and more than 90% for CCA and Least Absolute Shrinkage and Selection Operator Analysis (LASSO).
Choi et al., 2019 [27]	SSVEP in virtual reality (VR) environments	Pattern-reversal checkerboard stimulus (PRCS) and Grow/shrink stimulus (GSS): star pattern, luminance change and size in head-mounted displays (HMDs).	Cz, PO3, POz, PO4, O1, Oz, O2	GSS has higher accuracy than PRCS, but the visual comfort score is the same for both.
Mu et al., 2021 [28]	Multi-frequency (superimposing with OR and ADD)	Red LED with two 50% duty cycle square waves with the OR and ADD operator with frequencies of 7 and 9 Hz, 7 and 11 Hz, 7 and 13 Hz, 9 and 11 Hz, 9 and 13 Hz, and 11 and 13 Hz.	PO3, POz, PO4, O1, Oz, O2	Average accuracy of 70.83% on frequency superposition stimulation.
Stawicki and Volosyak, 2021 [30]	Steady State Motion visual evoked potentials (SSMVEPs)	Full-color circle (SSVEP, (SSMVEP1, SSMVEP2) and Checkerboard circle (SSMVEP3-5) with frequencies of 7.06, 7.50, 8.00, and 8.57 Hz.	Pz, P3, P4, P5, P6, PO3, PO4, PO7, PO8, Oz, O1, O2, O9, O10, POO1, POO2	Average accuracy between 97.22% and 100% and an average ITR between 15.42 and 33.92 bits/min.
Rekrut et al., 2021 [31]	Spinning Icons SSVEP	Spinning icons including check, arrow, box, cross, gear, icon check, icon email, icon PDF, icon spread, and icon text with frequencies of 7.5, 10, and 13 Hz.	Oz, P7, P3, Pz, P4, T7, Cz, T8, F3	Highest accuracy is 86% from cross SSMVEP followed by PDF icon with an accuracy of 75% (which is a remarkable result for a three-class classification problem with a chance level of 33.3%).

**Table 2:** Proposed flickering frequencies and patterns of SSVEP stimulus.

Flicker	Pattern	Flickering Frequency	
		Fundamental	Sub/Harmonics
1	Single	7 Hz	-
2	Single	13 Hz	-
3	Single	17 Hz	
4	Mixture	7 Hz	14 Hz
5	Mixture	13 Hz	6.5 Hz
6	Mixture	7, 13 Hz	-
7	Mixture	7, 17 Hz	-
8	Mixture	13, 17 Hz	-

**Table 3:** Results of the average classification accuracy of all participants, through different flicker patterns.

Flicker Patterns		Average Classification Accuracy (%)			
		SSVEP Detection Methods			
		PSD		CCA	
		Checkerboard	QR Code	Checkerboard	QR Code
1		90.9	89.3	84.9	87.3
2		88.0	90.6	89.3	91.2
3		85.9	91.8	85.3	89.5
4	^+^	83.7	90.2	87.1	90.5
5	^+^	84.9	93.4	91.2	94.4
6	^++^	87.9	90.5	87.3	92.3
7	^++^	84.7	89.3	89.5	91.8
8	^++^	87.1	88.0	90.5	93.0
Mean ± SD.		86.6 ± 2.32	90.4 ± 1.66	88.1 ± 2.34	91.2 ± 2.19

Note: ^+^ indicates the mixing of the fundamental and its harmonic frequency; ^++^ indicates the mixing of two fundamental frequencies.

**Table 4:** Results of average classification accuracy of SSVEP detection methods from different flicker patterns of the checkerboard and QR code patterns for each participant.

Participants	Average Classification Accuracy (%)			
	SSVEP Detection Methods			
	PSD		CCA	
	Checkerboard	QR Code	Checkerboard	QR Code
1	89.8	90.5	90.6	90.5
2	84.3	87.5	91.3	94.4
3	85.2	89.8	85.8	88.2
4	85.8	89.1	89.1	89.7
5	89.8	85.9	83.5	90.5
6	83.6	89.7	84.3	94.0
7	84.4	93.0	85.8	92.5
8	86.6	90.6	85.8	91.3
9	84.4	90.5	89.8	91.7
10	85.9	89.7	87.5	93.8
11	85.9	90.6	86.6	89.8
12	89.1	85.9	88.2	90.9
Mean ± SD.	86.2 ± 2.19	89.4 ± 2.06	87.4 ± 2.43	91.4 ± 1.91

## Data Availability

The data presented in this study are available upon request from the corresponding author.
